# Biomedical Semantics: the Hub for Biomedical Research 2.0

**DOI:** 10.1186/2041-1480-1-1

**Published:** 2010-03-31

**Authors:** Dietrich Rebholz-Schuhmann, Goran Nenadic

**Affiliations:** 1 European Bioinformatics Institute, Wellcome Trust Genome Campus, Hinxton, Cambridge, CB10 1SD, UK

## 

We would like to welcome you to *Journal of Biomedical Semantics *(JBMS), a new open-access, peer-reviewed inter-disciplinary journal that addresses novel theoretical and practical advances in biomedical semantics research. The main focus of JBMS is the development and integration of semantics resources into the biomedical research practice. The journal therefore aims at providing a forum for dissemination of achievements that have generated or used semantics resources to tackle challenges in biology and medicine, for example in genetics, medical treatments, development of therapeutics, translational medicine, food production and others.

## 1. The Rise of Biomedical Semantics

New discoveries in biology and medicine over the last two decades have been facilitated by large-scale data generation exercises as part of high-throughput experiments [1]. The observed data is maintained in ever growing scientific public databases as structured data, while novel scientific findings are reported in a semi- or un-structured form in the literature. Many of the past and present developments in biomedicine, in particular in computational biology and medical informatics, have focused on structured data management. At the same time, there has been a remarkable increase in the number and coverage of semantics resources and semantically annotated repositories to leverage biomedical knowledge from their structured and unstructured origins alike [2].

As part of the new developments, biomedical research is not relying only on local data and findings generated within individual laboratories, but is increasingly integrating shared, publicly available biomedical repositories. Furthermore, researchers exploit data resources from miscellaneous domains: for example, molecular biologists using toxicological data and medical scientists exploring data resources from molecular biology or environmental sciences. Altogether, biomedical research is moving beyond approaches utilizing "hand-crafted" hypotheses and inference techniques towards approaches that are trained on large-scale, semantically integrated biomedical data. For example, research in systems biology and systems medicine requires semantic integration of data across the biomedical domain to feed it into comprehensive and formalised models of living beings, utilising techniques from computer science, mathematics and engineering to analyse and predict biological and medical outcomes based on experimental and formal semantics descriptions.

Indeed, in recent years, we have been witnessing an evolution towards formalising biomedical knowledge in explicit domain models such as curated and annotated datasets, ontologies, pathways, semantic networks, etc [3]. The biomedical semantics *resourceome *now contains a large number of repositories. Biomedical ontologies [4], terminologies and controlled vocabularies (e.g. the Gene Ontology [5], MeSH [6], UMLS [7], ICD-10 [8], etc.), in particular, have been widely used for the integration of diverse scientific databases and for standardisation of information access through common but stringent logical definitions and constraints on semantic annotations of database content. Such semantic descriptors have been also used for detailed analyses and improvement of our understanding of known and prediction of yet un-explained biomedical processes.

In addition to the development of the semantics resourceome for structured data, researchers have been targeting the automated integration and exploitation of unstructured data, i.e. the scientific literature, to capture novel results and findings [9]. Information retrieved from semantics-driven literature mining needs to be integrated into existing knowledge repositories, thus becoming an integral part of a fully specified and interconnected knowledge space for biomedical research.

The need for formalised semantics is, of course, not specific to biomedicine. However, the unprecedented scale of biomedical data has put biomedical research into a forefront position to foster the development of leading-edge technologies such as linked data and the Semantic Web. We can already see a growing penetration of these technologies into the life science community, evident through many initiatives and conferences (e.g. Bio2RDF [10], BioHackathon [11], SWAT4LS [12]). In addition to institutionally-supported actions, there are various community-driven initiatives to annotate, organise and provide access to the whole spectrum of biomedical resources, from primary experimental data to data analysis workflows (e.g. myExperiment [13]) and secondary semantically-curated resources (e.g. ORegAnno [14]).

Management and maintenance of the semantics resourceome has already been recognised as one of the most challenging tasks that the community needs to address in order to facilitate our understanding of biological processes and predictions of functional behaviour. An interoperable and comprehensive semantic infrastructure is likely to facilitate data, knowledge and methodology re-use and integration, and thus encourage further developments in the field. Coupled with human- and machine-readable formal models and automated reasoning, the infrastructure can provide an environment for the next generation of biomedical research, one that will rely on a distributed semantic grid of biomedical data and knowledge. Some authors already refer to these developments as *Semantic Systems Biology *(and *Medicine*), where reasoning and conclusions are based on the full integration of biomedical knowledge [15]. Combined with semantics-driven data analysis workflow orchestration and distributed execution, the new framework for *in-silico *biomedical experimentation has the potential to add a new dimension to the way biomedical research is conducted [16].

One question that many biomedical semantics researchers often face is whether their research aims to create a virtual scientist, who would do automated investigations and will, eventually, replace real-world scientists. While such attempts have been explored [17], most of the current research in the domain of biomedical semantics is focused on the *facilitation *of large-scale, systematic and formalised representation and exploration of biomedical knowledge, where the main onus is still on an expert to mange and experience the scientific discovery process, but using a research environment that can cope with the complexity, dynamics and volume of biomedical knowledge.

Biomedical semantics research thus aims to bridge the gap between the data and knowledge that has been increasingly available and facilitate their use to support scientific exploration. Only full semantic integration of biomedical knowledge and experimental data can provide a means for scientists to model complex biological systems and thus improve our understanding of living organisms.

## 2. JBMS: towards the next generation of biomedical investigations

JBMS is primarily focused on the engineering, population and scientific exploitation of semantic resources in biomedicine. It encompasses the full variety of biomedical semantic resourceome (i.e. ontologies, taxonomies, terminologies, controlled vocabularies, annotated data, knowledge and service repositories, literature, reasoning systems, etc.) and their use in data and knowledge integration, mining, modelling, interpretation and exploitation in and for biomedical research. Examples of targeted scientific achievements include annotation and curation work of biomedical data resources, development of ontologies, inference of knowledge from publicly available data resources (e.g. exploiting data and text mining or reasoning techniques), and integration and exploitation of resources as part of the emerging Semantic Web solutions.

JBMS aims at publishing interdisciplinary research that crosses the traditional boundaries between the involved disciplines (i.e. biology, medicine, mathematics, computer science, bioinformatics) and areas (knowledge representation, information retrieval, data mining, reasoning and visualisation). The journal will therefore aim to attract contributions that are currently scattered over journals in different fields, and are typically focused on the perspective of a particular discipline (where the publication will be deposited), rarely addressing the entire complexity of biomedical semantic modelling and usage. The full scope of topics of interest is given on the journal's About page. The two main topic areas of the journal are:

### (1) Infrastructure for biomedical semantics

This area focuses on development of semantic resources and the underlying infrastructure, including data and knowledge representation models; provenance, curation, evolution, validation, evaluation and dissemination of semantic resources; semantic integration and mapping between data resources, biomedical Semantic Web, etc.

### (2) Exploitation of semantic resources

Typical examples would include using biomedical text and data mining for automated hypothesis generation; exploitation of the Semantic Web and linked data in biomedical research; large-scale automated analyses of biomedical data using ontology-driven reasoning for prediction of novel findings, etc.

JBMS aims to be in the forefront of scientific semantic publishing, and will work closely with both the publisher (BioMed Central) and research community to establish an infrastructure and best practice guidelines for developing and annotating semantic resources, including the publications themselves. We aspire to provide the authors and readers with the opportunities to semantically enrich the publications so that they could become part of an integrated semantics network of biomedicine.

We are very pleased to introduce the distinguished members of the Editorial Board. All members have significantly contributed to the research domains relevant to JBMS and will help to shape the development of the journal, providing both strategic guidelines and management of the peer-review process. The scientific interests of the Board members represent the broad range of topics that are relevant to JBMS, ranging from biology and medicine to bioinformatics, medical and health informatics, computer science and philosophy. We would like to express our gratitude to all of them for their engagement and help to establish and launch this journal.

The authors of the inaugural issue deserve our thanks and congratulations. The first manuscripts accepted for publication include contributions from the conference *Languages in Biology and Medicine 2009 *[18]. A special issue on biomedical ontologies will follow, featuring extended versions of the publications from the Bio-Ontology Special Interest Group meeting in 2009 [19].

We are also grateful to BioMed Central publishing company and its members of staff for the technical support and their encouragements to set up the journal, but also for leading the way in promoting the ideas and establishing research forums that cover emerging areas in biomedical sciences.

Altogether, we hope you will enjoy learning about the findings, challenges and solutions reported in JBMS, and that you will actively contribute to establishing biomedical semantics research as a key, next generation biomedical research area. We expect that the JBMS will become a hub for the dissemination of this type of research to the wider scientific community. We would therefore encourage all potential readers and authors to contribute their research work to JBMS and become part of this exciting research domain.

Dietrich Rebholz-Schuhmann and Goran Nenadic

Editors-in-Chief
